# Cultural adaptation, content validity and inter-rater reliability of the "STAR Skin Tear Classification System"^1^

**DOI:** 10.1590/0104-1169.3523.2537

**Published:** 2015

**Authors:** Kelly Cristina Strazzieri-Pulido, Vera Lúcia Conceição de Gouveia Santos, Keryln Carville

**Affiliations:** 2 MSc, RN, Instituto do Câncer do Estado de São Paulo "Octavio Frias de Oliveira", São Paulo, SP, Brazil; 3 PhD, Associate Professor, Escola de Enfermagem, Universidade de São Paulo, São Paulo, SP, Brazil; 4 PhD, Professor, Primary Health Care and Community Nursing, Silver Chain and Curtin University, Perth, WA, Australia

**Keywords:** Wounds and Injuries, Skin Aging, Validation Studies

## Abstract

**AIMS:**

to perform the cultural adaptation of the STAR Skin Tear Classification System into the Portuguese language and to test the content validity and inter-rater reliability of the adapted version.

**METHODS:**

methodological study with a quantitative approach. The cultural adaptation was developed in three phases: translation, evaluation by a committee of judges and back-translation. The instrument was tested regarding content validity and inter-rater reliability.

**RESULTS:**

the adapted version obtained a regular level of concordance when it was applied by nurses using photographs of friction injuries. Regarding its application in clinical practice, the adapted version obtained a moderate and statistically significant level of concordance.

**CONCLUSION:**

the study tested the content validity and inter-rater reliability of the version adapted into the Portuguese language. Its inclusion in clinical practice will enable the correct identification of this type of injury, as well as the implementation of protocols for the prevention and treatment of friction injuries.

## Introduction

Skin tear, as it is known internationally, is a traumatic wound resulting from friction or friction and shear, leading to the separation of the epidermis from the dermis or the separation of both from the underlying structures^(^ 1 ^)^. Fragile skin is commonly associated with frail and dependent people, or those with impaired mobility and nutrition, such as elderly people^(^ 1 ^)^, those in the terminal phase of life^(^ 2 ^)^ and newborns^(^ 3 ^)^.

The theme is little known in Brazil, where skin tears were called lacerations^(^ 4 ^)^. Although this seems to be mere semantics, the fact of not having it own nomenclature is an important barrier to the implementation of specific preventive measures, as well as appropriate treatment techniques. To use standardized language and a reliable classification instrument are fundamental for evaluating the wound and planning the care, as well as being essential for the development of studies and the use of their results in clinical practice^(^ 5 ^)^.

Payne and Martin were the first to propose the skin tear nomenclature and classification system^(^ 1 ^)^. Although this classification is the most widely used, its measurement properties have not been demonstrated^(^ 1 ^)^. As there was no universally accepted nomenclature or classification instrument^(^ 6 ^)^ Carville et al.^(7) ^redesigned the Payne and Martin instrument in the light of evidence-based practice. The result was the STAR Skin Tear Classification System (STAR), a simple and easy to use, yet comprehensive instrument, with confirmed content validity and inter-rater reliability, and standardized terms and definitions^(^ 7 ^)^.

The STAR consists of a treatment guide, classification system and glossary. The treatment guide has six topics related to the care of the wound and the surrounding skin. The classification system evaluates the presence/absence of the skin flap and its viability. It contains five photographs, each relating to a description of a skin tear category. Finally, on the back of the instrument there is a glossary that provides the tear skin definitions and technical terms related to the theme^(^ 8 ^-^ 9 ^)^.

Aiming for the systematization of the knowledge concerning this type of injury, the objective of this study was to carry out the cultural adaptation of the STAR (treatment guide, classification system and glossary) for the Portuguese language of Brazil, as well as to test its content validity and inter-rater reliability.

## Methods

To perform the cultural adaptation and test the content validity and inter-rater reliability of the STAR, authorization was sought and received from Prof. Carville, the STAR project coordinator, and Prof. Santos. The Research Ethics Committee of the University of São Paulo School of Nursing approved the project (Authorization No. 859/2009 / CEP-EEUSP) and the "Octavio Frias de Oliveira" Cancer Institute of São Paulo State (ICESP) approved the clinical application. All ethical aspects required for research involving human beings were respected. Informed consent was obtained from all participants prior to their enrollment in the study and they were assured anonymity in all aspects.

This study is a methodological study with a quantitative approach, in which the cultural adaptation was carried out and the measurement properties of content validity and inter-rater reliability were tested^(^ 10 ^-^ 11 ^)^. - The cultural adaptation was developed in three phases:

Translation: conversion of the STAR into Portuguese. Two translations of the STAR into Portuguese were performed: One by a Brazilian connected to the health area and another by a Brazilian layman. Both translators were fluent in English.

- Evaluation by a committee of judges: content validation of the Portuguese version(10). The committee of judges was composed of six Brazilian nurses (stomatherapy or dermatology specialists), fluent in English and knowledgeable of the concepts to be analyzed. Based on the analysis of semantic, idiomatic, cultural and conceptual equivalence(11), the judges initially analyzed the instrument as a whole, determining its scope, that is, whether each concept was adequately covered by the set of items and all dimensions were included. They also analyzed the individual items of the instrument, checking their clarity and relevance. Regarding clarity, the judges reviewed the wording of the items and whether they were written so that the concepts involved were understandable and adequately expressed. Regarding relevance or representativity, they evaluated whether the items really reflected the concepts involved, whether they were relevant and whether they were adequate to achieve the aims proposed. The judges also evaluated the wording of the items to ensure that the concepts could be understood by users and whether they reflected those originally proposed, based on knowledge of the pathophysiology of the injury, always having the freedom to suggest adjustments and improvements needed in each item. After receipt of the analysis of the judges, the authors examined the equivalence between them and the controversial aspects were discussed in order to reach a consensus. Concordance of 80% was adopted for the equivalence analysis between the evaluations. The researchers discussed the controversial aspects of these evaluations and obtained a Portuguese version, with validated content, which was back-translated.

- Back-translation: conversion of the Portuguese version into English. For Beaton and colleagues(11), this is a way to verify the adequacy of the adaptation and to detect translation inconsistencies. It was performed by two Brazilian lay translators different from those of the first translation step, who were ignorant of the aims of the translation. The back-translations were sent to Prof. Carville for the judgment of their equivalence with the original instrument.

Content validity was confirmed by the committee of judges, as described above; the inter-rater reliability was tested in two ways:

- Application of the Portuguese version using photographs: 107 Brazilian nurses who participated in the VIII Brazilian Congress of Stomatherapy (25 to 29 October 2009, Goiás) agreed to participate. The nurses were instructed to associate the descriptions of the skin tear categories of the Portuguese version, with five photographs of skin tears from the STAR, purposely placed out of the original order. It is noteworthy that these nurses were unaware of the STAR or other skin tear classification.

- Clinical application of the Portuguese version: The inter-rater reliability was verified by 20 nurses who classified skin tears in hospitalized patients, using the Portuguese version of the STAR. All 20 nurses who agreed to participate were Brazilian, members of the hospital nursing staff and claimed no knowledge of the STAR or other skin tear classification.

In this step, all patients hospitalized in the inpatient and intensive care units of the ICESP, 18 years or over, who agreed to participate, were submitted to the interview and visual inspection of the skin in order to identify the skin tears. Socio-demographic and clinical data were also collected from the medical records. A total of 10 beds per shift (morning, afternoon and evening) were evaluated on nine consecutive days, from 10 to 18 April 2010. No bed, patient or injury was repeated.

The skin tears were classified by the principle investigator (gold standard), according to the Portuguese version of the STAR. Consecutively, with the patient maintaining the same position, each nurse of the unit from which the patient originated independently performed their classification, also using the Portuguese version of the STAR.

**Figure 1 -:**
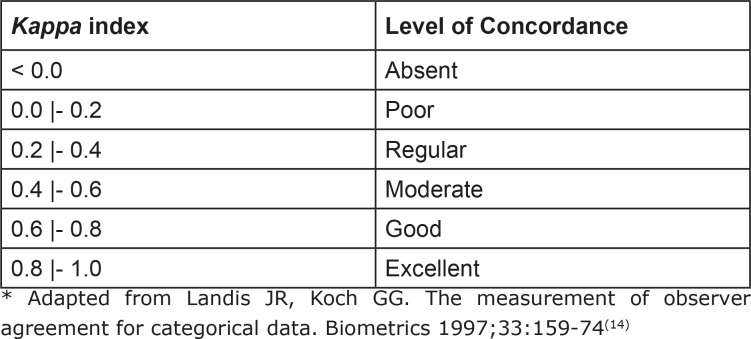
Eeighted kappa indices according to the level of concordance.

For the analysis of the socio-demographic and clinical data, measures of central tendency were used. The inter-rater reliability was evaluated by weighted kappa (wk). Negative values represent concordance lower than that expected due to chance or inconsistency of the test^(^ 12 ^-^ 13 ^)^. The concordance is excellent if wk>0.8 and poor if wk<0.2^(^ 14 ^)^. The assignment of the values related to the weights is subjective and depends on the context^(^ 13 ^)^. Figure 1 presents the interpretation of the distribution of the *wk* according to the level of concordance. Tests with a significance level less than 5% (p <0.05) were considered statistically significant.

## Results

The results are presented according to the steps outlined in the methods.

### Cultural adaptation

### Translation

Two Portuguese versions of the STAR were obtained. The translators reported difficulty with the words 'skin tear', 'bruising' and 'dusky'. Regarding the expression 'skin tear', there is no equivalent term in Portuguese and in relation to the terms 'bruising' and 'dusky', English - Portuguese bilingual dictionaries translate bruising as inflamed and dusky, as dark, opaque. Only the lay translator reported difficulty in translating the instrument, the other had experience in translating medical documents. The other differences were related to synonyms, verb tenses and prepositions, which did not compromise any equivalences.

### Evaluation by the Committee of Judges: 

For the analysis of semantic and idiomatic equivalence, the degree of concordance between the committee members was 81.8%. The judges also reported difficulties in adapting the expression 'skin tear'. Since there is no idiomatic or cultural equivalent in Brazil, they (83.3%) chose a literal translation. The lack of an equivalent expression in Portuguese and, considering that the expression chosen by the expert committee was not a good cultural equivalent and did not cover the entire contents of the English expression, the researchers assumed the responsibility of choosing the most appropriate expression, this being *lesões por fricção* (friction injuries). From now on friction injury will be used rather than skin tear.

### Back-translation

No problems were reported in either back-translations. All terms were back-translated identically to the original, with the only exception being 'dusky' translated as 'opaque', in one of the versions. This was sent to Prof. Carville , the author of the original instrument, who verified the excellent equivalence of both versions with the original.

### Inter-rater reliability

### Photographic set

Considering the five categories of friction injuries, each classified by 107 nurses, 535 observations were obtained.

Table 1 presents the frequency of errors and correct responses for each friction injury category.

**Table 1 -:** Errors and correct responses of respondents for each friction injury category of the Portuguese version, when applied with the photographic set. São Paulo, SP, Brazil, 2010

Friction injury categories	Errors			Correct responses			Total	
	n	%		n	%		n	%
Category 1a	56	52.3		51	47.7		107	100.0
Category 1b	70	65.4		37	34.6		107	100.0
Category 2a	65	60.7		42	39.3		107	100.0
Category 2b	51	47.7		56	52.3		107	100.0
Category 3	61	57.0		46	43.0		107	100.0
Total	303	56.6		232	43.4		535	100.0

The frequency of correct responses was lower than the errors in all categories except for 2b (56/52.3%). Categories 1b and 2a presented error frequencies exceeding 60.0% (70/65.4% and 65/60.7% respectively).

Table 2 presents the levels of concordance between the responses of the nurses and the template of the data collection instrument - photographs.

**Table 2 -:** Responses of nurses regarding the friction injury categories of the Portuguese version, when applied with the photographic set, according to levels of concordance (weighted kappa index). São Paulo, SP, Brazil, 2010

Level of Concordance	Level of Concordance (wk)	Number of responses	%
Absent	< 0.0	307	57.4
Poor	0.0 |- 0.2	-	-
Regular	0.2 |- 0.4	79	14.8
Moderate	0.4 |- 0.6	149	27.8
Good	0.6 |- 0.8	-	-
Excellent	0.8 |- 1.0	-	-
Total		535	100.0

Table 2 shows that, for the majority (307/57.4%) of the 535 responses, the level of concordance was absent and only 149 (27.8%) obtained a moderate level. The Portuguese version obtained a regular (wk = 0.286), although statistically significant (p<0.001), level of concordance.

### Clinical application

Of the 183 hospitalized patients at the time of data collection, 5 (2.7%) presented nine friction injuries. One patient who presented three friction injuries refused to participate and was excluded from the study. The mean age of the patients was 63.7 years (29 to 86 years); three were women, three were white, three were retired and three reported a family income of one minimum wage. Two had bowel cancer; two presented metastases and three had undergone surgical treatment.

Regarding the risk factors associated with friction injuries, 4 patients reported a history of friction injury, presented bruises or hematomas and edema in the extremities, rigidity and spasticity, impaired mobility, were dependent for basic daily living activities, were subject to transfers and repositioning, falls and knocks, and used invasive devices, adhesive bandages and a mean of 5.7 drugs/ person.

Considering that six friction injuries were identified, each classified by 5 nurses, 30 classifications were obtained.

Table 3 presents the frequency of errors and correct responses for each friction injury category.

**Table 3 -:** Errors and correct responses of the respondents for each friction injury category of the Portuguese 159 www.eerp.usp.br/rlae Strazzieri-Pulido KC, Santos VLCG, Carville K. version, related to the clinical application. São Paulo, SP, Brazil, 2010

Friction injury categories	Errors			Correct responses			Total	
	n	%		n	%		n	%
Category 1a	2	40.0		3	60.0		5	100.0
Category 1b	3	60.0		2	40.0		5	100.0
Category 2a	4	80.0		1	20.0		5	100.0
Category 2b	1	20.0		4	80.0		5	100.0
Category 3	4	40.0		6	60.0		10	100.0
Total	14	46.7		16	53.3		30	100.0

The frequency of correct responses was higher than that of the errors in categories 1a (3/60.0%), 2b (4/80.0%) and 3 (6/60.0%) and, as in the application with the photographic set, categories 1b and 2a presented higher frequencies of errors (3/60.0% and 4/80.0%).

Table 4 presents the levels of concordance between the responses of the nurses and the gold standard.

**Table 4 -:** Responses of the nurses regarding the friction injury categories of the Portuguese version, related to the clinical application, according to the levels of concordance (weighted kappa index). São Paulo, SP, Brazil, 2010

Level of Concordance	Level of Concordance (wk)	Number of responses	%
Absent	< 0.0	14	46.7
Poor	0.0 |- 0.2	-	-
Regular	0.2 |- 0.4	1	3.3
Moderate	0.4 |- 0.6	2	6.7
Good	0.6 |- 0.8	9	30.0
Excellent	0.8 |- 1.0	4	13.3
Total		30	100.0

The results in Table 4 show that for 14 (46.7%) of the 30 responses the level of concordance between the nurses and the gold standard was absent; only 13 (43.3%) presented good to excellent levels. The Portuguese version obtained moderate and statistically significant levels of concordance (wk = 0.596; p<0.001).

## Discussion

In the cultural adaptation, the difficulty that the translators and judges had with the expression 'friction injury' was highlighted. The authors considered the terms proposed to be vague and poorly associated with the original. The Portuguese term was selected by referring to the etiology of the wound and, resembling another culturally and technically familiar term - pressure ulcer, establishing the nomenclature according to its cause: friction injury. Although the literature recommends a meeting between judges in the absence of concordance, the authors judged themselves qualified to discuss the controversial aspects and reach the final version adapted to Portuguese.

Difficulties with the expression 'friction injury' chosen for the Portuguese version were not reported in the back-translations, confirming the adequacy of the choice and excellent association between the translated term and the original. Despite the lack of idiomatic and cultural equivalence, the expression demonstrated conceptual equivalence.

All these considerations led the researchers to consider that the content validity of the instrument adapted for Brazil was confirmed.

The inter-rater reliability was analyzed through two strategies: photographic, as in the original study^(^ 7 ^)^ and clinical^(^ 11 ^)^.

The worse performance of the instrument with the photographs can be attributed to the absence of a photographic set for comparison. The nurses correlated the five photographs of the instrument to their corresponding categories, therefore, there were no other photographs as the basis for comparison, unlike the original study by Carville et al.^(^ 7 ^)^. For the clinical application of the instrument, however, the nurses could associate the photographs of the adapted instrument with the wounds found on the patients. This detail can provide an advantage in the use of the instrument, expanding the explanations for the differences in the statistical analysis.

In the application of the original instrument, performed by Carville et al.^(^ 7 ^)^, 26 trained nurses classified 25 photographs of friction injuries. The concordance levels were below 65.0% for categories 1b, 2a and 2b. One reason for the low concordance was the lower quality of the photographs of these categories compared to the photographs of category 1 and 3. The application of the instrument exclusively with photographs constituted a limitation of the original study^(^ 7 ^)^.

In the present study, in addition to the absence of a photographic set for comparison, a negative impact due to the quality of the photographs was also observed. However, since the assembly of a photography database for comparison would require another validation work, not only of "content" (images) but also of the equivalence to the original photographs, which would constitute another study, it was decided to also perform the application of the instrument in the clinical practice. It was considered that, for the evaluation of wounds, the *in vivo* model provides the advantage of a three-dimensional evaluation, in addition to the possibility of the inspection and palpation of the wound in search of the skin flap. Pallor, opacity or darkening of the flap and surrounding skin, as well as fragility, edema, discoloration or bruising around the wound are easier to perceive *in vivo* than in photographs.

For the construction of the original instrument, the concordance obtained for each friction injury category was much higher than in the present study, in both applications (photographic and clinical), ranging from 83.0% to 97.0%, with 93.0% for the photographic set^(^ 7 ^)^. Only category 1b obtained concordance below 90.0%, with 85.0%.

In the present study, category 1b did not present good performance in either application. The percentage of correct responses was only 34.6% in the application with photographs and slightly better (40.0%) in the clinical practice. In the version of the instrument published in 2007^(8) ^and used in this study, this category was represented by an injury with non-invasive sutures. As well as the low quality of this photograph compromising the results obtained in the STAR project, this probably also compromised the concordance obtained here.

Regarding the difference between the results of both studies, another aspect to be considered is related to the fact that the original authors^(^ 7 ^)^ verified the inter-rater reliability after training the nurses involved. In the present study, at no time were the nurses trained to use the adapted instrument. Furthermore, in Brazil, the expression 'friction injury' lacks an idiomatic or cultural equivalent. Thus, these two factors as considered to be limitations of the study.

The degree of cicatrization of the wounds may also have contributed to the results obtained. The categories that presented the highest frequency of correct responses, 2b (4/80.0%) and 1a (3/60.0%), during the clinical application, had wounds of two and three days, respectively. Probably the fact that they were recent facilitated the evaluation of conditions of the skin flap and the wound bed. Conversely, the categories with the lowest frequency of correct responses, 2a (1/20.0%) and 1b (2/40.0%) had wounds of seven days. The skin flaps were found to be well integrated into the bed of the wounds, making the evaluation difficult for the less attentive observers.

For friction injury category 2a, the evaluation was only possible because the wound bed showed traces of the skin flap. Similarly, the flap of friction injury category 1b presented only a slight darkening, which easily passed unnoticed by two nurses.

Despite friction injury category 3 already being seven days into the healing process, the absence of the skin flap may have facilitated the evaluation, contributing to the higher frequency of correct responses (6/60.0%). In the first version of the original instrument, the concordance obtained for category 3 was above 90.0% and one hypothesis is that the evaluation of the skin flap is more difficult than that of the wound bed^(^ 7 ^)^.

It can be said that the moderate levels of concordance obtained (*wk* = 0.596) in relation to the clinical application of the Portuguese version, confirmed the inter-rater reliability.

The cultural adaptation of the STAR and the performance of tests to validate its contents and inter-rater reliability are the first initiatives to arouse the interest of Brazilian healthcare professionals regarding the problem involving friction injuries. It is hoped that this study will inspire other initiatives in order to identify environments, situations and people at risk, as well as risk factors, incidence and prevalence of these injuries, characteristic to Brazil. In addition, the inclusion of the evaluation of these types of wounds in daily clinical practice will enable the implementation of protocols for their prevention and adequate treatment. Visualizing the future, it is expected that friction injuries will receive the same attention, dedication and care that is currently given to pressure ulcers, and that this will also be seen as an indicator of the quality of the healthcare services.

Regarding the present study, although two measurement properties of the instrument were confirmed, the validation process does not end here. Validation is an ongoing process. The more evidence that can be gathered related to the instrument measuring what it purports to measure, the greater the confidence in the results of its use^(^ 11 ^)^.

Other limitations in addition to those mentioned here should be highlighted. It was not possible to make any comparison, beyond that carried out with the original version, since the instrument has not been validated in other languages. The lack of publications related to friction injuries and studies involving other classification instruments for these wounds also made it impossible to compare the findings obtained in this study with those of other authors.

An important limitation refers to the institutional sample. The application of the instrument in clinical practice was restricted to one institution and a single type of patient, limiting the sample size. Conversely, this led to an unexpected situation: that of finding this injury in young patients and not only in elderly people, as reported in the literature. Aging has been appointed as the main factor involved in the pathophysiology of this injury, however, the fragility of the skin, also present at the other extreme of age, at the end of life and in some conditions such as cachexia, characterize it as the preponderant factor^(^ 2 ^)^.

It is recommended that new clinical applications are carried out in other types of institutions, and that nurses are trained in the use of the version adapted to Portuguese, through protocols for the prevention and treatment of wounds, developed by specialists.

The STAR Classification System - Friction Injury is available in the Digital Library of Theses and Dissertations of USP (www.teses.usp.br/teses/disponiveis/7/7139/tde.../Kelly_Pulido_ME.pdf).

## Conclusion

This study led to the conclusion that the measurement properties of content validity and of inter-rater reliability of the STAR *Skin Tear Classification System* instrument were confirmed for its version adapted to the Portuguese of Brazil and, therefore, the Portuguese version of the STAR Classification System - Friction Injury can be used in clinical practice.
